# Early Estimated Glomerular Filtration Rate Trajectories After Kidney Transplant Biopsy as a Surrogate Endpoint for Graft Survival in Late Antibody-Mediated Rejection

**DOI:** 10.3389/fmed.2022.817127

**Published:** 2022-04-21

**Authors:** Anita Borski, Alexander Kainz, Nicolas Kozakowski, Heinz Regele, Johannes Kläger, Robert Strassl, Gottfried Fischer, Ingrid Faé, Sabine Wenda, Željko Kikić, Gregor Bond, Roman Reindl-Schwaighofer, Katharina A. Mayer, Michael Eder, Markus Wahrmann, Susanne Haindl, Konstantin Doberer, Georg A. Böhmig, Farsad Eskandary

**Affiliations:** ^1^Department of Nephrology and Dialysis, Medical University Vienna, Vienna, Austria; ^2^Department of Pathology, Medical University Vienna, Vienna, Austria; ^3^Division of Clinical Virology, Department of Laboratory Medicine, Medical University Vienna, Vienna, Austria; ^4^Department of Blood Group Serology and Transfusion Medicine, Medical University Vienna, Vienna, Austria; ^5^Department of Urology, Medical University Vienna, Vienna, Austria

**Keywords:** surrogate end point validation, antibody-mediated allograft rejection, landmark analysis, donor-specific anti HLA antibodies, allograft loss, estimated glomerular filtration rate (eGFR), fine and gray model

## Abstract

**Background:**

Late antibody-mediated rejection (ABMR) after kidney transplantation is a major cause of long-term allograft loss with currently no proven treatment strategy. Design for trials testing treatment for late ABMR poses a major challenge as hard clinical endpoints require large sample sizes. We performed a retrospective cohort study applying commonly used selection criteria to evaluate the slope of the estimated glomerular filtration rate (eGFR) within an early and short timeframe after biopsy as a surrogate of future allograft loss for clinical trials addressing late ABMR.

**Methods:**

Study subjects were identified upon screening of the Vienna transplant biopsy database. Main inclusion criteria were (i) a solitary kidney transplant between 2000 and 2013, (ii) diagnosis of ABMR according to the Banff 2015 scheme at >12 months post-transplantation, (iii) age 15–75 years at ABMR diagnosis, (iv) an eGFR > 25 mL/min/1.73 m^2^ at ABMR diagnosis, and (v) a follow-up for at least 36 months after ABMR diagnosis. The primary outcome variable was death-censored graft survival. A mixed effects model with linear splines was used for eGFR slope modeling and association of graft failure and eGFR slope was assessed applying a multivariate competing risk analysis with landmarks set at 12 and 24 months after index biopsy.

**Results:**

A total of 70 allografts from 68 patients were included. An eGFR loss of 1 ml/min/1.73 m^2^ per year significantly increased the risk for allograft failure, when eGFR slopes were modeled over 12 months [HR 1.1 (95% CI: 1.01–1.3), *p* = 0.020] or over 24 months [HR 1.3 (95% CI: 1.1–1.4), *p* = 0.001] after diagnosis of ABMR with landmarks set at both time points. Covariables influencing graft loss in all models were histologic evidence of glomerulonephritis concurring with ABMR as well as the administration of anti-thymocyte globulin (ATG) at the time of transplantation.

**Conclusion:**

Our study supports the use of the eGFR slope modeled for at least 12 months after biopsy-proven diagnosis of late ABMR, as a surrogate parameter for future allograft loss. The simultaneous occurrence of glomerulonephritis together with ABMR at index biopsy and the use of ATG at the time of transplantation–likely representing a confounder in pre-sensitized recipients–were strongly associated with worse transplant outcomes.

## Introduction

Late antibody-mediated rejection (ABMR) after kidney transplantation is a major cause of long-term allograft loss and a difficult-to-treat disease entity, since its detection is frequently hampered by a clinically indolent onset, even in the presence of meanwhile often established routine longitudinal donor-specific antibody (DSA) testing and protocol biopsy programs (1, 2). This may result in irreversible chronic glomerular damage and fibrosis consistent with chronic ABMR (cABMR) at the time of diagnosis (3). Furthermore, treatment strategies are limited to date and especially in the presence of cABMR, no therapeutic agent has been shown to interfere with the course of kidney functional decline and allograft loss rates when compared to the standard of care i.e., the optimization of maintenance immunosuppression (2, 4). Recently, promising treatment strategies such as interleukin-6 (IL-6) blockade with monoclonal antibodies tocilizumab or clazakizumab were tested in small clinical trials and currently clazakizumab undergoes intense investigation in the up-to-date largest phase III study ever conducted in patients with ABMR (IMAGINE trial, NCT03744910) (5, 6).

One of the major hurdles for the design of such a trial is the difficulty of defining a valid and feasible endpoint (7). The gold standard of demonstrating improvement in graft survival was shown to be an unrealistic endpoint in trials addressing late ABMR, since in general the overall number of eligible patients with a potentially reversible disease course is low and the timespan from diagnosis until graft loss does often last over many years (8). This would therefore require large sample-sizes, only realizable when embedded within international multicenter trials (9). The definition of adequate surrogate endpoints has therefore been proposed and the use of the slope of estimated glomerular filtration rate (eGFR) is widely accepted as such a measure, next to other upcoming promising novel endpoints (7, 9–12).

In this context we investigated whether eGFR slopes within 12–24 months after a biopsy showing ABMR would deliver sufficient information to evaluate its association with graft survival in a clinically meaningful way that might aid at identifying suitable patients to be enrolled into clinical trials. This retrospective study was conducted by screening the Vienna transplant biopsy database for late biopsies formally fulfilling morphologic criteria for ABMR and by applying in- and exclusion criteria commonly used in trials addressing ABMR in order to create a highly granular cohort. Historic sera were re-evaluated for the presence of DSA at the time of biopsy, and we applied appropriate statistical models with purposeful selection of covariables to model eGFR slopes and to assess their impact on graft survival in patients diagnosed with late active ABMR.

## Materials and Methods

### Study Design

This is a retrospective study designed to assess the correlation of the eGFR slope within the first 12–24 months after biopsy-proven late active ABMR with graft survival. We defined pre- and post-biopsy time points with pre-specified deviation windows for kidney function measurements (Supplementary Table 1). Our patient cohort was derived by searching our electronic transplant biopsy database (Department of Pathology, Medical University Vienna) for lesions compatible with ABMR and by applying the Banff 2015 criteria (13). Once a biopsy formally fulfilled histomorphologic and immunohistochemical criteria of active or chronic active ABMR following the Banff 2015 scheme, the following inclusion and exclusion criteria were applied:

### Inclusion Criteria

- Kidney transplant recipient of a solitary live or deceased donor kidney transplanted between January 1, 2000 and July 31, 2013.
- Kidney transplantation at least 12 months prior to index biopsy.
- Diagnosis of biopsy-proven ABMR according to Banff 2015 criteria on or before July 31, 2014.
- Age 15–75 years at the time of diagnosis of ABMR.
- At least two longitudinal measurements of serum creatinine within the first year prior to or at diagnosis of ABMR, including baseline creatinine at the time of diagnosis, and at least two measurements of serum creatinine post-diagnosis of ABMR.
- Minimum of 3 years follow-up after diagnosis of ABMR to ascertain allograft status.

### Exclusion Criteria

- Recipient of a multi-organ transplant.
- First diagnosis of active ABMR after July 31, 2014.
- eGFR < 25 ml/min/1.73 m^2^ at the time of diagnosis of active ABMR to exclude patients with a high likelihood of timely graft loss.

Patients who were transplanted but not followed at our or at an associated center were excluded. Since many biopsies were carried out before the implementation of routine Luminex testing at our center, which was available since 2009, and in order to verify serological presence of anti-HLA donor-specific antibodies (DSA) at the time of index biopsy, we collected frozen historical sera from different in-house sources when available and subjected them to pooled single-antigen bead testing. We defined a final analysis cohort of 70 allografts composed of 55 allografts with antibody-verified ABMR at the time of biopsy and 15 allografts highly suspicious for ABMR, but without definitive proof of DSA at the time of biopsy. We included these 15 biopsies in the analysis based on recent Banff updates with less emphasis on imperative DSA confirmation (13, 14). However, as provided in the Supplementary Tables 1–5 and Supplementary Figures 1–4, the 55 allografts with verified DSA were also analyzed separately to assess consistency of our results.

This study was approved by the institutional ethics committee of the Medical University Vienna (EK1104/2019) and was carried out in compliance with the Good Clinical Practice Guidelines, principles of the Declaration of Helsinki 2008, and the Declaration of Istanbul.

### Data Extraction and Laboratory Measurements

Demographic variables and laboratory measurements were collected using the hospital’s patient management software and medical records at the transplant outpatient clinic of the Department of Nephrology and Dialysis, Division of Medicine III at the Medical University of Vienna. Estimated glomerular filtration rate (eGFR) was assessed using the seven-variable MDRD equation provided that data on serum albumin and blood urea nitrogen (BUN) were available. Otherwise, we used the four-variable MDRD equation or, for one pediatric patient, the Schwartz equation was used (15, 16). We chose the MDRD formula since the majority of our patients already had reduced eGFR at the time of index biopsy.

### Human Leukocyte Antigen Antibody Detection

We used LABscreen Single Antigen assays (One Lambda, A Thermo Fisher Scientific Brand, Canoga Park, CA, United States) for the characterization of anti-HLA reactivity patterns according to the manufacturer. Testing was performed retrospectively on frozen sera or plasma obtained close to or at the time of biopsy. To counteract complement interference, serum samples were treated with 10 mM EDTA (17). Donor-specificity was determined using serological, low- or high-resolution donor/recipient HLA typing methods for HLA-A, -B, -Cw, -DR, -DQ and -DP, whichever was available. A mean fluorescence intensity (MFI) value of 1,000 was used as the threshold for positivity, below 1,000 only clear epitope reactivity patterns on several beads > 500 MFI were counted as positive. The immunodominant DSA was called according to the bead that revealed the highest MFI.

### Biopsies

Histomorphologic lesions and immunohistochemistry (C4d) were assessed on formalin-fixed paraffin-embedded sections. We applied the Banff 2015 classification to diagnose ABMR using the following lesion criteria: glomerulitis (g), peritubular capillaritis (ptc), transplant glomerulopathy (cg), and C4d (BI-RC4D; Biomedica, Vienna, Austria). All included biopsies were either for clinical indication (*n* = 58) or study protocol biopsies performed within the BORTEJECT trial (*n* = 12) (18).

We also included cases of ABMR with concurrent glomerulonephritis (GN), taking histomorphologic criteria besides endocapillary hypercellularity and/or basal membrane contours for the diagnosis of ABMR into account.

### Outcome Analysis

The pre-defined outcome of this study was death-censored graft survival defined as return to permanent dialysis, re-transplantation or transplant nephrectomy, whichever occurred first. Secondary outcomes were overall graft survival and patient death. Patients alive at the date of last known follow-up or at the end of study were right censored. All patients were followed up until July 31, 2017 (end of study).

### Statistics

Previously defined patient characteristics at the time of transplantation and at the time of index biopsy were summarized using descriptive statistics. Variables were tested for statistically independent distribution between patients who experienced graft loss during the study period versus patients who did not. For categorical variables, the Chi-Squared test was used while for metric or ordinally scaled variables, the Mann-Whitney *U*-test was used. For visual inspection of overall- and death-censored graft survival the Kaplan-Meier method was used. When analyzing subgroups, group comparison was done using the Log-rank test.

Change of eGFR across time was modeled as a linear spline by a linear mixed effects (LME) model. Two different LME models were calculated, the first one stretching to 12 months after index biopsy (time point 12) and the second one to 24 months after biopsy (time point 24), to account for different study periods that would be acceptable in therapeutic intervention trials. In both models, the intercept was defined at the time of index biopsy (time point 0) and the slope before biopsy was calculated starting at 12 months before index biopsy (time point -12). Linear splines were used in the LME models, avoiding a step in the curve at the intercept and allowing different slopes before and after biopsy. Statistical significance of the eGFR slopes was examined by the one-sample *t*-test.

#### Modeling the Relationship Between Estimated Glomerular Filtration Rate Slope and Time-to-Event Data

The association between change in eGFR and risk of graft failure was assessed with the multivariate competing risk proportional hazard regression model from Fine and Gray, where graft loss represents the event of interest and death the competing risk (19). For all covariables sub-distribution hazards are given. Patients alive with a functioning graft at the date of last known follow-up or at the end of study were censored. The proportional hazards assumption was checked by examining Schoenfeld type residuals. In case of non-proportionality we added a term of the concerned variable multiplied with the logarithm of time for correction.

Two different analyses predicting future graft loss were performed: One beginning at 12 months after index biopsy, using the eGFR slope modeled over -12 to 12 months, and the other model beginning at 24 months after index biopsy, using the eGFR slope modeled over -12 to 24 months. Landmarks were set at the time points 12 and 24 to exclude events that happened already before collection of model information was completed, and since prognostic statements cannot be based on information available in the future (20). The eGFR slope before index biopsy and the eGFR slope after index biopsy were both engaged as covariables. Other clinically meaningful covariables were included into the model such as deceased donor type, use of lymphocyte depleting agent as induction agent (used in pre-sensitized patients at our center), the occurrence of a glomerulonephritis in addition to ABMR within the biopsy, TCMR concurrent with ABMR, C4d-positivity in PTC and triple immunosuppression at index biopsy.

For all analyses SAS 9.4 for Windows (Cary, NC, United States), IBM SPSS Statistics Version 24 (IBM, Armonk, NY, United States) and GraphPad Prism 9.1.1 (GraphPad Software, San Diego, CA, United States) was used. A two-sided p-value of less than 0.05 was considered statistically significant.

## Results

### Study Flow and Patient Demographics

**FIGURE 1:**
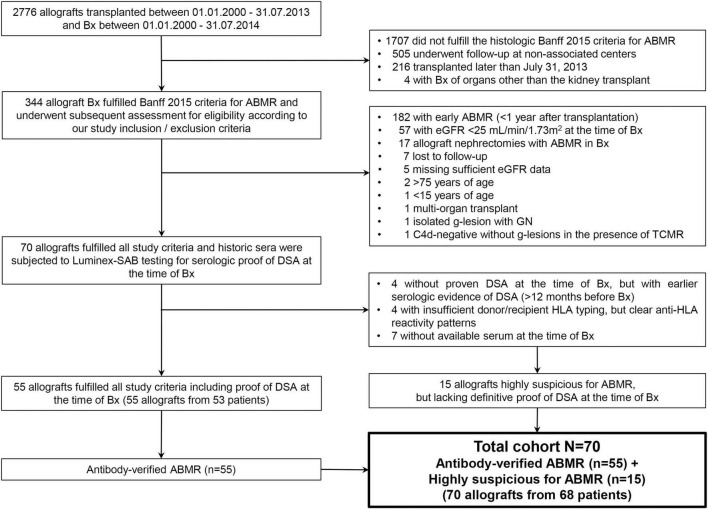
Flowchart of the biopsy and patient selection process. After strictly applying all inclusion and exclusion criteria historical sera were searched for in order to carry out collective SAB testing as some biopsies dated back until the year 2000, long before Luminex testing was available at our center. The total cohort consisted of 70 biopsies from 68 patients (two biopsies came from repeat kidney transplants from the same two patients).

As depicted in Figure 1, we extracted 2,776 biopsies that fulfilled the primary screening criteria for transplantation period, timing of biopsy and center follow-up. Overall, 344 biopsies formally fulfilled the histopathologic criteria for Banff 2015 ABMR and were further assessed for detailed in- and exclusion criteria. A total cohort of 70 allografts from 68 patients was analyzed.

#### Variables at the Time of Transplantation

Patient baseline demographics at the time of transplantation are provided in Table 1. Median recipient age was 46 years (IQR 30–54) and 28 patients (40%) were female. Prior kidney transplantation had occurred in 22 (31%) of patients and median highest CDC-PRA was 10% (IQR 3–46). The median donor age was 48 years (IQR 37–57) and 84% of included patients received a deceased donor transplant.

**TABLE 1:** Variables recorded at transplantation.

Parameter	Total cohort (*n* = 70)	Graft loss (*n* = 31)	No graft loss (*n* = 39)	*p*-value
**Variables recorded at transplantation**				
Recipient age, years, median (IQR)	46 (30–54)	41 (28–53)	51 (35–56)	0.142
Female sex, *n* (%)	28 (40)	9 (29)	19 (49)	0.095
Primary diagnosis				
Glomerulonephritis, *n* (%)	18 (26)	10 (32)	8 (21)	n.a.
Vascular nephropathy, *n* (%)	2 (3)	0 (0)	2 (5)	n.a.
Diabetes, *n* (%)	3 (4)	0 (0)	3 (8)	n.a.
Polycystic kidney disease, *n* (%)	9 (13)	7 (23)	2 (5)	n.a.
Hypertension, *n* (%)	6 (9)	2 (6)	4 (10)	n.a.
Other, *n* (%)	32 (46)	12 (39)	20 (51)	n.a.
Prior kidney transplant, *n* (%)	22 (31)	14 (45)	8 (21)	0.027
Pre-sensitized (CDC-PRA ≥ 40% or DSA), *n* (%)*^a^*	26 (37)	11 (35)	15 (39)	0.798
CDC-PRA				
Highest,% median (IQR)	10 (3–46)	10 (0–76)	10 (4–30)	0.892
Latest,% median (IQR)	1 (0–17)	1 (0–22)	0 (0–15)	0.767
Preformed anti-HLA DSA [17/70 (24%) were tested pre-Tx], *n* (%)*^b^*	15 (88)	3 (18)	12 (71)	n.a.
HLA class I, *n* (%)*^b^*	4 (24)	1 (6)	3 (18)	n.a.
HLA class II, *n* (%)*^b^*	3 (18)	0 (0)	3 (18)	n.a.
Both classes, *n* (%)*^b^*	5 (29)	3 (18)	2 (12)	n.a.
Class unknown, *n* (%)*^b^*	3 (18)	0 (0)	3 (18)	n.a.
Peri-transplant (induction) therapy, *n* (%)*^c^*	33 (47)	13 (42)	20 (51)	0.436
IA + ATG/anti-IL-2 antibody or ATG/Muromonab-CD3, *n* (%)	24 (34)	12 (39)	12 (31)	0.487
Anti-IL-2 antibody as single induction agent, *n* (%)	9 (13)	1 (3)	8 (21)	0.032
Delayed graft function, *n* (%)	20 (29)	8 (26)	12 (31)	0.648
Donor age, years, median (IQR)	48 (37–57)	51 (40–58)	45 (35–57)	0.242
Deceased donor, *n* (%)	59 (84)	26 (84)	33 (85)	0.932

*^a^Before 2009, pre-sensitized patients were defined as having a CDC-PRA ≥ 40%.*

*^b^Refers to percent of patients that underwent Luminex testing before transplantation, which was available since 2009 in our center.*

*^c^Four patients had a positive CDC-XM and underwent peritransplant-XM conversion with immunoadsorption according to our center-protocol. ATG, anti-thymocyte globulin; CD3, cluster of differentiation 3; CDC-PRA, complement-dependent cytotoxicity panel-reactive antibodies; DSA, donor-specific antibody; HLA, human leukocyte antigen; IA, immunoadsorption; IQR, interquartile range; IL-2, interleukin-2; n.a., not applicable; Tx, transplantation; XM, crossmatch.*

With respect to differences in baseline variables, patients who experienced graft loss (*n* = 31, 44%) vs. no graft loss had a tendency toward more frequent prior kidney transplantation (45 vs. 21%, *p* = 0.027) and were less frequently administered induction therapy with an anti-IL-2 antibody (3 vs. 21%, *p* = 0.032).

Baseline variables for the antibody-verified cohort (*n* = 55) are provided in Supplementary Table 2. Here we found numerically more frequent prior kidney transplantations in patients who experienced graft loss vs. no graft loss (42 vs. 19%, *p* = 0.071) and their CDC-PRA was significantly higher [Median highest CDC-PRA 14% (IQR 10–77) vs. 7% (0–22), *p* = 0.022 and median latest CDC-PRA 4% (0–55) vs. 0% (0–13), *p* = 0.025].

#### Variables at the Time of Index Biopsy

As provided in Table 2, the median recipient age was 49 years (IQR 36–58) and the median time from transplantation to biopsy was 34 months (IQR 19–75). With respect to biopsy-cause, we found that 83% of patients were biopsied for clinical indication and 17% were biopsied for the presence of DSA without clinical deterioration. When comparing patients experiencing graft loss vs. no graft loss, we found that patients with graft loss were more often biopsied for clinical cause (94 vs. 74%, *p* = 0.034) and had a lower median baseline eGFR [35 (IQR 28–39) vs. 45 mL (39–54), *p* < 0.001] respectively.

**TABLE 2:** Variables recorded at index biopsy.

Parameter	Total cohort (*n* = 70)	Graft loss (*n* = 31)	No graft loss (*n* = 39)	*p*-value
**Variables recorded at index biopsy**				
Age, median (IQR)	49 (36–58)	47 (35–54)	54 (40–59)	0.113
Time Tx to iBx (months), median (IQR)	34 (19–75)	56 (19–76)	32 (22–65)	0.624
Baseline eGFR (mL/min/1.73 m^2^), median (IQR)	40 (33–48)	35 (28–39)	45 (39–54)	<0.001
Index biopsy for clinical cause (vs. DSA +), *n* (%)	58 (83)	29 (94)	29 (74)	0.034
Rise in serum creatinine, *n* (%)	20 (29)	11 (35)	9 (23)	0.254
Onset of or rise in proteinuria, *n* (%)	22 (31)	9 (29)	13 (33)	0.700
Both, *n* (%)	16 (23)	9 (29)	7 (18)	0.273
Anti-HLA DSA at iBx*^a^*				
HLA class I DSA only, *n* (%)*^a^*	17 (31)	9 (38)	8 (26)	0.352
HLA class II DSA only, *n* (%)*^a^*	22 (40)	8 (33)	14 (45)	0.375
HLA class I and II DSA, *n* (%)*^a^*	16 (29)	7 (29)	9 (29)	0.991
MFI_sum of all detected DSA, median (IQR)*^a^*	15,726 (6,044–25,732)	15,230 (6,967–34,219)	16,104 (4,416–22,754)	0.585
Immunodominant anti-HLA DSA at iBx*^a^*				
HLA class I*^a^*	20 (37)	11 (46)	9 (29)	0.157
HLA class II*^a^*	34 (62)	12 (50)	22 (71)	0.157
MFI_max, median (IQR)*^a^*	11,733 (5,505–16,403)	13,684 (6,967–16,780)	9,235 (4,229–16,575)	0.642
*De novo* anti-HLA DSA [14/70 (20%) with known DSA specificities pre-Tx]*^a,b^*	7 (50)	1 (7)	6 (43)	n.a.
*De novo* HLA II DSA in pre-Tx DSA + patient, *n* (%)*^a,b^*	5 (36)	1 (7)	4 (29)	n.a.
*De novo* HLA II DSA in pre-Tx DSA- patient, *n* (%)*^a,b^*	2 (14)	0 (0)	2 (14)	n.a.
No *de novo* DSA in pre-Tx DSA + patient, *n* (%)*^a,b^*	7 (50)	2 (14)	5 (36)	n.a.
Triple immunosuppression, *n* (%)	53 (76)	23 (74)	30 (77)	0.791
Tacrolimus-based, *n* (%)	31 (44)	13 (42)	18 (46)	0.724
Cyclosporine A-based, *n* (%)	18 (26)	10 (32)	8 (21)	0.264
mTORi-based, *n* (%)	4 (6)	0 (0)	4 (10)	0.066
Dual immunosuppression, *n* (%)	16 (23)	8 (26)	8 (21)	0.600
No steroids, *n* (%)	7 (10)	3 (10)	4 (10)	0.936
No MMF/MPA/Azathioprine, *n* (%)	7 (10)	3 (10)	4 (10)	0.936
No CNI/mTORi, *n* (%)	2 (3)	2 (6)	0 (0)	0.108
CNI monotherapy, *n* (%)	1 (1)	0 (0)	1 (3)	0.369
Medication non-adherence reported by patient, *n* (%)	6 (9)	2 (6)	4 (10)	0.572
Anti-rejection treatment following iBx, *n* (%)	43 (61)	21 (68)	22 (56)	0.333
Steroids, *n* (%)	19 (27)	13 (42)	6 (15)	0.013
ATG or IVIG, *n* (%)	2 (3)	1 (3)	1 (3)	0.869
IA or PLEX ± steroids ± IVIG, *n* (%)	11 (16)	6 (19)	5 (13)	0.456
Rituximab + IVIG, *n* (%)	3 (4)	0 (0)	3 (8)	0.114
Bortezomib, *n* (%)	8 (11)	1 (3)	7 (18)	0.054

*^a^Numbers refer to the 55 patients with verified DSA at the time of biopsy provided in Supplementary Table 2 (Graft loss: n = 24, no graft loss n = 31), respectively.*

*^b^Refers to percent of patients that underwent Luminex testing before transplantation, which was available since 2009 at our center and where the specificities of pre-Tx DSA were documented. In three patients with verified DSA before transplantation, specificities were not documented. ATG, anti-thymocyte globulin; iBx, index biopsy; CNI, calcineurin inhibitor; DSA, donor-specific antibody; HLA, human leukocyte antigen; IA, immunoadsorption; IQR, interquartile range; IVIG, intravenous immunoglobulins; MFI, mean fluorescence intensity; MMF, mycophenolate mofetil; MPA, mycophenolic acid; mTORi, inhibitor of mammalian target of rapamycin; n.a., not applicable; PLEX, plasma exchange; Tx, transplantation.*

When analyzing anti-HLA reactivities we found that 31% had DSA against HLA class I only, 40% against HLA class II only and 29% against both HLA class I and II (Table 2). Median sum of all DSA MFI was 15,726 (IQR 6,044-25,732) and 62% of immunodominant DSA (DSA with the highest MFI) were directed against HLA class II. The median MFI of the immunodominant DSA was 11,733 (IQR 5,505–16,403). DSA-specificities are depicted in Supplementary Figure 1. The detection of *de novo* DSA was limited by the relatively small proportion with available pre-Tx DSA status. Seventeen patients had undergone pre-Tx Luminex testing, of which 15 patients were DSA-positive (including three cases where anti-HLA allele-specificity was not recorded) and two patients were DSA-negative. We found that seven of the 14 patients (50%) with known anti-HLA-specificities (12 DSA-positive cases, two DSA-negative cases) had developed *de novo* DSA, of which all were directed against HLA class II (Table 2). Specificities of *de novo* DSA are provided in Supplementary Table 3. In patients who experienced graft loss vs. no graft loss we found no statistical differences regarding any DSA specificity or strength (Table 2 and Supplementary Table 2).

With respect to immunosuppression, at the time of biopsy 76% of patients were on triple immunosuppression, 23% on dual immunosuppression and 1% was on CNI monotherapy. There were no differences between patients with graft loss vs. without graft loss regarding immunosuppressive medication. Overall, 43 (61%) of patients were treated with any anti-rejection treatment following biopsy. Of the 19 (27%) patients who had received intravenous steroids as anti-rejection treatment, we found a significant difference between patients who experienced graft loss vs. without graft loss (42 vs. 15%, *p* = 0.013, Table 2).

### Biopsy Characteristics

Histomorphologic and immunohistochemical results are provided in Table 3. The majority of biopsies (71%) fulfilled the Banff 2015 criteria for chronic/active ABMR whereas 29% fulfilled the criteria for acute/active ABMR. With respect to complement activation detected in immunohistochemistry we found that 26 biopsies (37%) had a positive linear C4d staining in PTC. Twenty-nine (41%) of biopsies showed concurrent TCMR, 14 cases presented with concurrent GN (20%) and in four cases (6%) thrombotic microangiopathy was present. In biopsies with transplant glomerulopathy we found that patients who experienced graft loss had a higher median cg score compared to patients without graft loss [cg score 3 (IQR 1–3) vs. 1 (0–3), *p* = 0.026].

**TABLE 3:** Index biopsy results.

Parameter	Total cohort (*n* = 70)	Graft loss (*n* = 31)	No graft loss (*n* = 39)	*p*-value
**Index biopsy results**				
Microcirculation inflammation (g > 0 ± ptc > 0), *n* (%)	67 (96)	29 (94)	38 (97)	0.425
g score, median (IQR)	2 (1–2)	2 (1–2)	2 (1–2)	0.859
ptc score, median (IQR)	2 (1–2)	2 (1–2)	2 (1–2)	0.342
g + ptc score, median (IQR)	3 (2–4)	3 (2–4)	3 (2–4)	0.859
Transplant glomerulopathy (cg > 0), *n* (%)	53 (76)	26 (84)	27 (69)	0.156
cg score, median (IQR)	2 (1–3)	3 (1–3)	1 (0–3)	0.026
Linear C4d + in PTC, *n* (%)	26 (37)	11 (35)	15 (38)	0.919
C4d score, median (IQR)	0 (0–2)	0 (0–1)	0 (0–2)	0.898
Histologic criteria of acute/active ABMR, *n* (%)	20 (29)	7 (23)	13 (33)	0.323
Histologic criteria of chronic/active ABMR, *n* (%)	50 (71)	24 (77)	26 (67)	0.323
Concurrent TCMR, *n* (%)	29 (41)	14 (45)	15 (38)	0.572
Borderline lesion, *n* (%)	18 (26)	9 (29)	9 (23)	n.a.
IA or IB, *n* (%)	6 (9)	3 (10)	3 (8)	n.a.
IIA, *n* (%)	4 (6)	1 (3)	3 (8)	n.a.
Chronic TCMR, *n* (%)	1 (1)	1 (3)	0 (0)	n.a.
Concurrent GN, *n* (%)	14 (20)	9 (29)	5 (13)	0.092
IgA nephropathy, *n* (%)	7 (10)	5 (16)	2 (5)	n.a.
Immune-complex GN (e.g., MPGN), *n* (%)	6 (9)	4 (13)	2 (5)	n.a.
Membranous GN, *n* (%)	1 (1)	0 (0)	1 (3)	n.a.
Thrombotic microangiopathy, *n* (%)	4 (6)	3 (10)	1 (3)	0.203

*ABMR, antibody-mediated rejection; cg, transplant glomerulopathy; g, glomerulitis; GN, glomerulonephritis; IQR, interquartile range; MPGN, membranoproliferative glomerulonephritis; n.a., not applicable; ptc, peritubular capillaritis; PTC, peritubular capillaries; TCMR, T cell-mediated rejection.*

### Overall, Death-Censored and Patient Survival Since Transplantation and Index Biopsy

**FIGURE 2:**
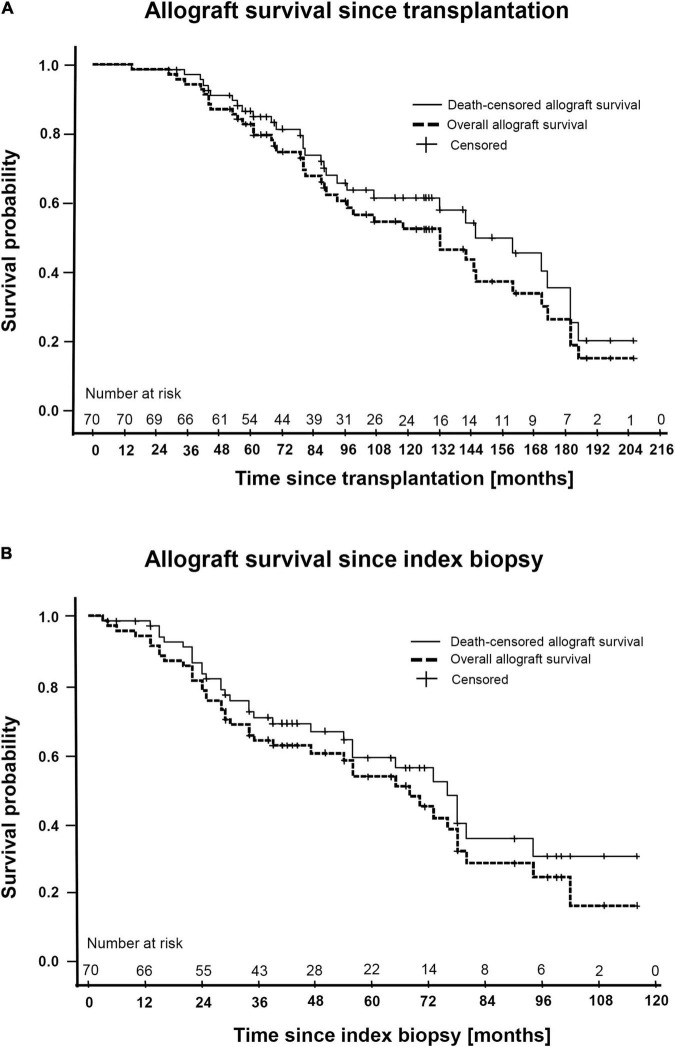
Kaplan-Meier plots of the total cohort (*N* = 70) including overall- and death-censored allograft survival since transplantation **(A)** and since index biopsy **(B)**.

The median follow-up time until graft loss, patient death or end of study was 88 months (IQR 61–132) after transplantation and 41 months (27–67) after index Bx (iBx). Unadjusted overall allograft survival at 1, 2, 3, 5, and 10 years after index iBx was 93, 80, 64, 53, and 15%, and 97, 83, 71, 59, and 29% for death-censored allograft survival, respectively. The median time until overall graft loss after transplantation was 132 months and 68 months after iBx, whereas median time until death-censored graft loss after transplantation was 160 months and 76 months after iBx. Patient survival at 1, 2, 3, 5, and 10 years after transplantation was 100, 96, 94, 88, and 55%, while after iBx patient survival was 94, 93, 90, 84, and 55%, respectively. Kaplan-Meier curves for overall allograft and death-censored allograft survival since transplantation and since iBx are provided in Figures 2A,B, patient survival since transplantation and iBx is depicted in Supplementary Figures 2A,B.

### Analysis of Estimated Glomerular Filtration Rate Slopes Before and Early After Index Biopsy

**FIGURE 3:**
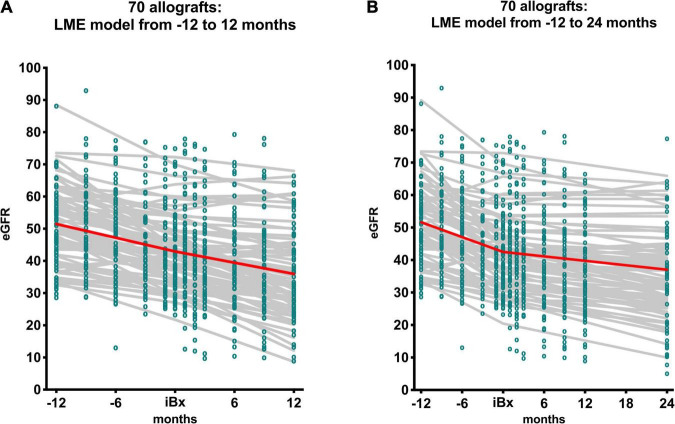
It shows the single eGFR measurements (green dots), the individual eGFR trajectories (gray solid lines) as well as the overall trajectory (red solid line) of the total cohort (*N* = 70). The linear mixed effects model (LME) was carried out over a course of 2 years **(A)** from −12 to 12 months with intercept at time of iBx and over the course of 3 years **(B)** from −12 to 24 months with intercept at the time of iBx. The shown overall trajectory is based on the fixed effects of the LME, whereas the individual trajectories also include the random effects.

The dynamics of eGFR trajectories spanning from 12 months before iBx until either 12 or 24 months after iBx were inspected applying a linear mixed effects (LME) model. Figures 3A,B depicts the single point eGFR measurements as well as the individual slopes and the mean overall eGFR slope from either −12 before to 12 months after iBx (Figure 3A) or from −12 before to 24 months after iBx (Figure 3B) for the total cohort. Linear splines were introduced to avoid a step at the transition from pre-iBx to post-iBx eGFR course. Supplementary Figures 3A,B provides both graphs for the antibody-verified cohort. Interestingly, in either observation period, from −12 to 12, and–even more pronounced–from −12 to 24 months post-iBx we observed a moderation of the eGFR decline in the post-iBx course. This observation was confirmed in the antibody-verified cohort. Table 4 and Supplementary Table 4 list the values for the eGFR intercept, the slope pre-iBx and the slope post-iBx from both LME models for the total cohort and the antibody-verified cohort accounting for fixed and random effects. Both, the total cohort and the antibody-verified cohort had well comparable eGFR intercepts at iBx. We detected a statistically highly significant overall decline of the eGFR slope in all models, that revealed a slight flattening of the post-iBx slope (*p* < 0.001). For example, the slope in the total cohort changed from pre-iBx −9.2 mL/min/1.73 m^2^ (95% CI: −12.1 to −6.2) to post-iBx −5.5 mL/min/1.73 m^2^ (95% CI: −7.3 to −3.6) in the 24-month LME model (Table 4, *p* < 0.001).

**TABLE 4:** eGFR slopes.

Parameters	Estimate	95% confidence		p-value
		interval		
Total cohort (*n* = 70)		Lower	Upper	
−12 to 12 months LME model				
Intercept at iBx [ml/min/1.73 m^2^]	42.3	40.1	45.8	<0.001
eGFR slope pre-iBx [ml/min/1.73 m^2^]	−8.5	−11.4	−5.6	<0.001
eGFR slope post-iBx [ml/min/1.73 m^2^]	−6.9	−9.8	−4.2	<0.001
−12 to 24 months LME model				
Intercept at iBx [ml/min/1.73 m^2^]	42.5	39.6	45.4	<0.001
eGFR slope pre-iBx [ml/min/1.73 m^2^]	−9.2	−12.1	−6.2	<0.001
eGFR slope post-iBx [ml/min/1.73 m^2^]	−5.5	−7.3	−3.6	<0.001

*iBx, index biopsy; eGFR, estimated glomerular filtration rate; LME, linear mixed effects model.*

Since our cohort contained a substantial amount (61%, Table 2) of patients who had received anti-rejection treatment after iBx showing ABMR, we wanted to assess whether this impacted on eGFR slopes. Therefore, we calculated an LME including anti-rejection treatment as fixed effect. We found no significant interaction of anti-rejection treatment with pre- as well as post-iBx eGFR slopes (Supplementary Table 5).

### The Impact of Early Estimated Glomerular Filtration Rate Slopes After Index Biopsy on Graft Loss

**FIGURE 4:**
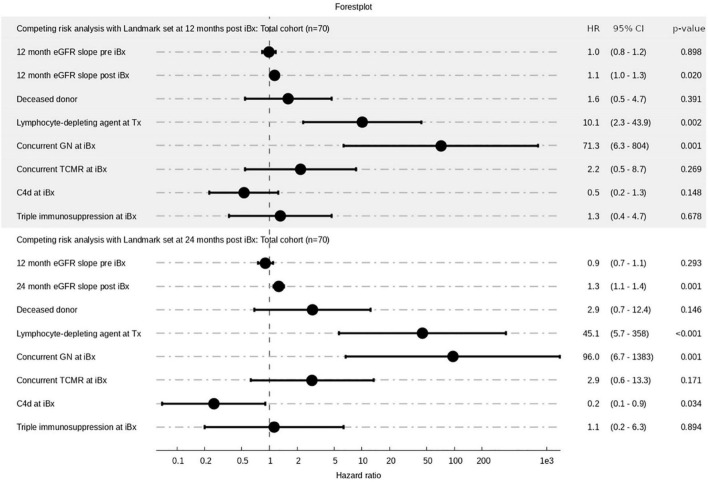
Forest plots for the total cohort (*N* = 70) with landmarks set at 12 or 24 months after iBx to avoid immortal time bias. Hazard ratios (HR’s) and their corresponding 95% confidence intervals (95% CI) are shown on the right. HR of 1 shows indifference of the variable regarding risk for allograft loss. GN, glomerulonephritis; TCMR, T cell-mediated rejection.

We applied a multivariate Cox proportional hazards regression model accounting for the competing risk of death to assess the impact of the pre-iBx (−12 months) and early post-iBx (12 or 24 months) eGFR slope on graft loss. To avoid immortal time bias we set a landmark at 12 months when looking at the 12 months post-iBx eGFR slope and at 24 months when evaluating the 24 months post-iBx eGFR slope. Figure 4 and Supplementary Figure 4 depict Forest plots of the total cohort and the antibody-verified cohort. We found in both models that the 12 months pre-iBx eGFR slope was not associated with allograft loss, whereas the 12 months and the 24 months post-iBx eGFR slope were significantly associated with graft loss. The hazard ratio (HR) for the 12 months post-iBx slope was 1.1 (95% CI: 1.0–1.3) reflecting a 10% risk increment with each 1 mL/min/1.73 m^2^ eGFR decline per year (*p* = 0.020). For the 24 months post-iBx eGFR slope we recorded a 30% risk increment for allograft loss with each 1 mL/min/1.73 m^2^ eGFR decline per year (HR: 1.3, 95% CI: 1.1–1.4, *p* = 0.001), respectively. Other variables with significant HRs in both models were the use of a lymphocyte depleting agent at Tx and the finding of a GN concurrent to ABMR. At inspection the covariable concurrent GN revealed violation of the proportional hazards assumption and was therefore corrected for as described in the Methods section. Nevertheless, a high HR of 71.3 (12 months post-iBx) and 96.0 (24 months post-iBx) with corresponding wide 95% CI (6.3–804 and 6.7–1383, *p* = 0.001) remained after correction in both models, possibly reflecting the deleterious effect of this less frequent biopsy-finding on allograft loss, and was therefore kept in the model.

## Discussion

We conducted this study to elucidate whether early eGFR slopes, from 12 up to 24 months after biopsy, could serve as a surrogate for future allograft loss in patients diagnosed with late ABMR. The search for a “minimally clinically meaningful difference” with respect to eGFR slopes as an accepted trial endpoint in kidney transplantation is ongoing and several recent studies have addressed this issue (8, 9, 21–23). In trials studying chronic kidney disease, eGFR slopes were recently accepted as a surrogate endpoint by the FDA, but their value in late ABMR remains elusive (12).

We evaluated early eGFR slopes with respect to their association with future allograft loss using LME models and analyzed them together with clinically meaningful covariables applying Cox regression and accounting for the competing risk of death. Landmarks excluding all events before their setpoint were placed at the end of the observation periods of interest to avoid overfitting of our model. Here we show, that an eGFR decline of only 1 mL/min/1.73 m^2^ per year within 12 to 24 months after biopsy with late ABMR was associated with an elevated risk of 10% (12-month slope) to up to 30% (24-month slope) for future allograft loss. Our findings thus implicate, that certain cutoffs in early post-iBx eGFR decline in patients with late ABMR might be defined as surrogate endpoint for allograft loss in future interventional trials. This might be of interest, since our aim was to specifically identify the value of early eGFR slopes in patients late after transplantation, whereas other studies have focused on eGFR decline within the earlier periods after transplantation as a surrogate endpoint for graft survival (24).

Very recently, Irish et al. (9) have carried out a retrospective multicenter study as part of a modeling exercise, applying a joint model for longitudinal data to the 12-month eGFR slope after a Bx with late active ABMR in order to simulate what impact different scenarios of eGFR slope-stabilization would have on predicted graft survival. The authors elegantly demonstrated that a stabilization of the eGFR slope of 30, 50, or 75%, based on the mean 12-month post-Bx slope, would lead to significantly improved graft survival after 2, 3, 4, and 5 years. Their concept might help to guide sample size calculations for the design of adaptive clinical trials in ABMR, based on putative eGFR slope-improvements potentially mediated by study drug interventions (9).

Our finding that the mean pre-iBx eGFR slope showed a steeper decline compared to the post-iBx eGFR slope, but was not significantly associated with graft survival, was unexpected. The reason for the smoothening of the eGFR slope after biopsy is unclear, but the effect of kidney function stabilization after biopsy was also shown in large studies such as the DeKAF Study, that next to other study questions also investigated the course of troubled kidney transplants with no intervention (25). Explanations for this finding in our study might be adaptation of baseline immunosuppression after the diagnosis of late ABMR, enhanced medical adherence or effects of anti-rejection treatment on ABMR concurring with a substantial rate of TCMR cases (41%) in our cohort. Although the majority of these concurring TCMR cases were borderline lesion (26%), we have tried to account for this by including TCMR in our multivariate model.

Our study also revealed other factors being differently distributed between patients who experienced graft loss compared to patients who did not experience graft loss within our observation period. Most factors associated with graft loss were reflecting a high likelihood of pre-sensitization, such as having received a prior kidney transplant or the use of ATG as induction agent. Others have found that graft survival in patients with *de novo* DSA was worse compared to patients with preformed DSA (26). In our cohort this analysis is precluded by the fact that before Luminex testing in 2009 only patients with a CDC-PRA ≥ 40% received induction with ATG, while patients with a CDC-PRA < 40% that nowadays might reveal a significant DSA when tested with Luminex did either receive an anti-IL-2 antibody or no induction therapy at all.

Another covariable that was significantly associated with graft loss was the occurrence of GN. This finding is not surprising, since it has already been shown, that the occurrence of a GN alone after transplantation is associated with reduced graft survival (27–29). However, in our model this covariables did not fulfill the proportional hazards assumption, which we corrected for, and resulted in a very high HR with an associated wide 95% confidence interval. Our explanation for this lies within the very high event rate after biopsy in an overall small subset of patients with this biopsy finding, thereby leaving only few cases in the later observation period at risk (30). In our opinion, this covariable is of importance when selecting patients for a clinical trial in ABMR, since on one hand their eGFR slope often shows a rapid decline and the future event of allograft loss is highly likely, but on the other hand it is unclear if any ABMR treatment will also have an impact on the course of a recurrent or *de novo* GN, making this concurrent diagnosis questionable for inclusion into interventional trials in ABMR.

Strengths of our study are the rigorous inclusion and exclusion criteria and the bottom-down selection process from > 2,500 biopsies spanning over two decades, that allowed us to build a representative cohort in the field of late ABMR. Furthermore, we set our focus on the verification of DSA at the time of biopsy and by testing historic sera, we were able to generate a highly granular and well-characterized patient cohort. Also, our collective included patients who were administered various different anti-rejection treatment modalities, which in clinical reality is a frequent situation when including patients into trials for late ABMR. Most of these treatments included the proteasome inhibitor bortezomib and the CD20 antibody rituximab for which it has already been shown that treatment of late ABMR is unsuccessful (18, 31).

To address the retrospective nature of this study with all the known causes for bias, we applied a well-designed statistical model that included various carefully selected covariables, accounted for competing risks, and set landmarks in order to account for immortal time bias. The latter also precluded the model to use event data within the period of the early eGFR course after biopsy to avoid overconfident outcome prediction.

Limitations of our study are the retrospective design, which makes it hard to decipher the unclear impact of ABMR management in patients from earlier transplant eras, when treatment algorithms were widely missing. Also, the change of Banff classification over time and the focus on different entities such as C4d could have led to different therapeutic decisions in these patients. Surprisingly, in our 24-month, but not in our 12-month eGFR slope model we found that C4d-positivity was not associated with a higher risk for graft loss, which is in contrast with other studies, but in our study might be explained by the fact that these patients could have undergone a higher post-Bx surveillance by the treating physicians and received more intense, and–in our cohort–also widely varying treatments (32). Other important limitations are the single-center design and a missing validation cohort. Lastly, we decided to also include patients, where no antibody-verification was possible at the time-of-biopsy, which reflects the fact that some ABMR cases may occur that miss the presence of classic anti-HLA DSA. Even though the majority of these cases in our study were mostly due to incomplete HLA typing of either donor or recipient, the histologic picture of ABMR could potentially also have been mediated by non-HLA DSA or NK cell-mediated missing-self processes (1, 33, 34).

In conclusion, our study is in line with the study by Irish et al. (9), showing that the early eGFR slope after biopsy-confirmed late ABMR can offer a valid surrogate endpoint for future allograft loss. We also detected GN concurrent with ABMR and the use of ATG at the time of transplantation, reflecting pre-sensitization status at our center, to be associated with a high risk of allograft loss. Future prospective data on eGFR slopes from large multicenter studies such as the IMAGINE trial (NCT03744910) may be used to further validate the early eGFR slope after biopsy-proven late ABMR for its implementation as a robust endpoint in clinical trials in transplantation.

## Data Availability Statement

The raw data supporting the conclusions of this article will be made available by the authors, without undue reservation.

## Ethics Statement

The studies involving human participants were reviewed and approved by Ethics committee of the Medical University of Vienna. Written informed consent from the participants’ legal guardian/next of kin was not required to participate in this study in accordance with the national legislation and the institutional requirements.

## Author Contributions

AB, AK, GAB, and FE designed the study, collected the data, analysed the data, and wrote the primary draft of the manuscript. HR, NK, JK, RS, GF, IF, SW, ŽK, GB, RR-S, KM, ME, MW, SH, and KD provided the materials needed for analysis, collected the data, and critically revised the manuscript. FE, GB, and GAB provided funding for the project. All authors agreed to the final submission of the manuscript.

## Conflict of Interest

The authors declare that the research was conducted in the absence of any commercial or financial relationships that could be construed as a potential conflict of interest.

## Publisher’s Note

All claims expressed in this article are solely those of the authors and do not necessarily represent those of their affiliated organizations, or those of the publisher, the editors and the reviewers. Any product that may be evaluated in this article, or claim that may be made by its manufacturer, is not guaranteed or endorsed by the publisher.

## Supplementary Material

Supplementary Figure 1 Bar charts with percentages of the HLA class I and class II antigens that the corresponding DSA were directed against. Since some patients had > 1 DSA within either HLA class, percentages do not perfectly match with the given percentages of class I and/or class II DSA at the time of iBx in Table 2 and Supplementary Table 2.

Supplementary Figure 2 Kaplan-Meier plots of the total cohort (*N* = 70) for patient survival since transplantation **(A)** and since index biopsy **(B)**.

Supplementary Figure 3 It shows the single eGFR measurements (green dots), the individual eGFR trajectories (gray solid lines) as well as the overall trajectory (red solid line) of the antibody-verified cohort (*N* = 55). The linear mixed effects model (LME) was carried out over a course of 2 years **(A)** from −12 to 12 months with intercept at time of iBx and over the course of 3 years **(B)** from -12 to 24 months with intercept at the time of iBx. The shown overall trajectory is based on the fixed effects of the LME, whereas the individual trajectories also include the random effects.

Supplementary Figure 4 Forest plots for the antibody-verified cohort (*N* = 55) with landmarks set at 12 or 24 months after iBx to avoid immortal time bias. Hazard ratios (HR’s) and their corresponding 95% confidence intervals (95% CI) are shown on the right. GN, glomerulonephritis; TCMR, T cell-mediated rejection.

## Abbreviations

ABMR: antibody-mediated rejection
ATG: anti-thymocyte globulin
Bx: biopsy
CD: cluster of differentiation
CDC: complement-dependent cytotoxicity
CI: confidence interval
CNI: calcineurin inhibitor
DSA: donor-specific antibody
EDTA: ethylenediaminetetraacetic acid
eGFR: estimated glomerular filtration rate
GN: glomerulonephritis
HLA: human leukocyte antigen
IA: immunoadsorption
iBx: index biopsy
IL: interleukin
IQR: interquartile range
IVIG: intravenous immunoglobulin
LME: linear mixed effects
MDRD: modification of diet in renal disease
MFI: mean fluorescence intensity
MMF/MPA: mycophenolate-mofetil/mycophenolic acid
mTORi: inhibitor of mammalian target of rapamycin
PLEX: plasma exchange
PRA: panel-reactive antibodies
PTC: peritubular capillaries
SAB: single antigen bead
TCMR: T cell-mediated rejection
Tx: transplantation
XM: crossmatch.
