# Supplementary material for: Early Estimated Glomerular Filtration Rate Trajectories After Kidney Transplant Biopsy as a Surrogate Endpoint for Graft Survival in Late Antibody-Mediated Rejection
Source: Front Med (Lausanne). 2022 Apr 21;9:817127. doi: 10.3389/fmed.2022.817127 (PMC9069161; doi:10.3389/fmed.2022.817127)
Supplement: Supplementary file 1 [file Data_Sheet_1.docx]

**Supplementary Tables**

**Supplementary Table S1**

| **Pre-specified timepoints** | | **Laboratory test window** | |
| --- | --- | --- | --- |
| **month** | **day** | **Deviation window (days)** | **day** |
| -12 | -365 | ± 60 | -425 to -305 |
| -9 | -274 | ± 30 | -304 to -244 |
| -6 | 1-83 | ± 30 | -213 to -153 |
| -3 | -91 | ± 30 | -121 to -61 |
| -1 | -30 | ± 14 | -16 to -44 |
| 0 | 0 | - 10 | -10 to 0 |
| 1 | 30 | ± 14 | 16 to 44 |
| 2 | 61 | ± 14 | 47 to 75 |
| 3 | 91 | ± 14 | 77 to 105 |
| 6 | 183 | ± 30 | 153 to 213 |
| 9 | 274 | ± 30 | 244 to 304 |
| 12 | 365 | ± 60 | 305 to 425 |
| 24 | 730 | ± 60 | 670 to 790 |
| 36 | 1096 | ± 60 | 1036 to 1156 |
| 48 | 1461 | ± 60 | 1401 to 1521 |
| 60 | 1826 | ± 60 | 1766 to 1886 |

**Supplementary Table S2**

| **Parameters**  **Antibody-verified ABMR cohort (n=55)** | **Total**  **(n=55)** | **Graft loss**  **(n=24)** | **No graft loss (n=31)** | **p-value** |
| --- | --- | --- | --- | --- |
| **Variables recorded at transplantation** |  |  |  |  |
| Recipient age, years, *median (IQR)* | 44 (28-54) | 41 (27-51) | 49 (29-55) | 0.197 |
| Female sex*, n (%)* | 22 (40) | 7 (29) | 15 (48) | 0.149 |
| Primary diagnosis |  |  |  |  |
| Glomerulonephritis*, n (%)* | 15 (27) | 9 (38) | 6 (19) | n.a. |
| Vascular nephropathy*, n (%)* | 2 (4) | 0 | 2 (7) | n.a. |
| Diabetes*, n (%)* | 3 (6) | 0 | 3 (10) | n.a. |
| Polycystic kidney disease*, n (%)* | 6 (11) | 4 (17) | 2 (7) | n.a. |
| Hypertension*, n (%)* | 5 (9) | 2 (8) | 3 (10) | n.a. |
| Other*, n (%)* | 24 (44) | 9 (38) | 15 (48) | n.a. |
| Prior kidney transplant*, n (%)* | 16 (29) | 10 (42) | 6 (19) | 0.071 |
| Pre-sensitized (CDC-PRA ≥ 40% or DSA), *n (%)^a^* | 22 (40) | 9 (38) | 13 (42) | 0.739 |
| CDC-PRA |  |  |  |  |
| Highest, % *median (IQR)* | 10 (3-46) | 14 (10-77) | 7 (0-22) | 0.022 |
| Latest, % *median (IQR)* | 1 (0-16) | 4 (0-55) | 0 (0-13) | 0.025 |
| Preformed anti-HLA DSA [17/55 (31%) were tested pre-Tx], *n (%)^b^* | 15 (88) | 3 (18) | 12 (71) | n.a. |
| HLA class I, *n (%)^b^* | 4 (24) | 1 (6) | 3 (18) | n.a. |
| HLA class II, *n (%)^b^* | 3 (18) | 0 (0) | 3 (18) | n.a. |
| Both classes, *n (%)^b^* | 5 (29) | 3 (18) | 2 (12) | n.a. |
| Class unknown, *n (%)^b^* | 3 (18) | 0 (0) | 3 (18) | n.a. |
| Peri-transplant (induction) therapy*, n (%)^c^* | 27 (49) | 10 (42) | 17 (55) | 0.333 |
| IA+ATG/anti-IL-2 antibody or ATG/Muromonab-CD3, *n (%)* | 21 (38) | 10 (42) | 11 (36) | 0.640 |
| Anti-IL-2 antibody as single induction agent, *n (%)* | 6 (11) | 0 (0) | 6 (19) | 0.022 |
| Delayed graft function*, n (%)* | 17 (31) | 6 (25) | 11 (36) | 0.404 |
| Donor age, years, m*edian (IQR)* | 48 (33-57) | 51(38-57) | 45 (27-58) | 0.484 |
| Deceased donor*, n (%)* | 45 (82) | 20 (83) | 25 (81) | 0.798 |
| **Variables recorded at index biopsy** |  |  |  |  |
| Age, *median (IQR)* | 48 (34-56) | 46 (33-53) | 53 (37-57) | 0.161 |
| Time Tx to iBx, (months), *median (IQR)* | 31 (19-66) | 39 (19-73) | 29 (19-52) | 0.599 |
| Baseline eGFR (mL/min/1.73m^2^), *median (IQR)* | 39 (33-50) | 35 (28-39) | 47 (36-54) | <0.001 |
| iBx for clinical cause (vs. DSA+), *n (%)* | 43 (78) | 22 (92) | 21 (68) | 0.033 |
| Rise in serum creatinine, *n (%)* | 17 (31) | 9 (38) | 8 (26) | 0.352 |
| Onset of or rise in proteinuria, *n (%)* | 16 (29) | 9 (38) | 7 (23) | 0.227 |
| Both, *n (%)* | 10 (18) | 4 (17) | 6 (19) | 0.798 |
| Anti-HLA DSA at iBx |  |  |  |  |
| HLA class I DSA only*, n (%)* | 17 (31) | 9 (38) | 8 (26) | 0.352 |
| HLA class II DSA only*, n (%)* | 22 (40) | 8 (33) | 14 (45) | 0.375 |
| HLA class I and II DSA*, n (%)* | 16 (29) | 7 (29) | 9 (29) | 0.991 |
| MFI_sum of all detected DSA, *median (IQR)* | 15,726  (6,044-25,732) | 15,230  (6,967-34,219) | 16,104  (4,416-22,754) | 0.585 |
| Immunodominant anti-HLA DSA at iBx |  |  |  |  |
| HLA class I | 20 (37) | 11 (46) | 9 (29) | 0.157 |
| HLA class II | 34 (62) | 12 (50) | 22 (71) | 0.157 |
| MFI_max, *median (IQR)* | 11,733  (5,505-16,403) | 13,684  (6,967-16,780) | 9,235  (4,229-16,575) | 0.642 |
| *De novo* anti-HLA DSA  [14/55 (25%) with known DSA specificities pre-Tx]^a,b^ | 7 (50) | 1 (7) | 6 (43) | n.a. |
| *De novo* HLA II DSA in pre-Tx DSA+ patient*, n (%)***^a,b^** | 5 (36) | 1 (7) | 4 (29) | n.a. |
| *De novo* HLA II DSA in pre-Tx DSA- patient*, n (%)***^a,b^** | 2 (14) | 0 (0) | 2 (14) | n.a. |
| No *de novo* DSA in pre-Tx DSA+ patient*, n (%)***^a,b^** | 7 (50) | 2 (14) | 5 (36) | n.a. |
| Triple immunosuppression*, n (%)* | 40 (73) | 17 (71) | 23 (74) | 0.781 |
| Tacrolimus-based*, n (%)* | 26 (47) | 10 (42) | 16 (52) | 0.464 |
| Cyclosporine A-based*, n (%)* | 12 (22) | 7 (29) | 5 (16) | 0.246 |
| mTORi-based*, n* *(%)* | 2 (4) | 0 | 2 (6) | 0.205 |
| Dual immunosuppression*, n* *(%)* | 14 (26) | 7 (29) | 7 (23) | 0.578 |
| No steroids*, n* *(%)* | 7 (13) | 3 (13) | 4 (13) | 0.965 |
| No MMF/MPA / Azathioprine*, n* *(%)* | 6 (11) | 3 (13) | 3 (10) | 0.739 |
| No CNI / mTORi*, n* *(%)* | 1 (2) | 1 (4) | 0 (0) | 0.251 |
| CNI monotherapy*, n* *(%)* | 1 (2) | 0 | 1 (3) | 0.375 |
| Medication non-adherence reported by patient*, n* *(%)* | 5 (9) | 2 (8) | 3 (10) | 0.863 |
| Rejection treatment*, n (%)* | 35 (64) | 15 (63) | 20 (65) | 0.877 |
| Steroids*, n (%)* | 13 (24) | 8 (33) | 5 (16) | 0.136 |
| ATG or IVIG*, n (%)* | 2 (4) | 1 (4) | 1 (3) | 0.853 |
| IA or PLEX ± steroids ± IVIG*, n (%)* | 9 (16) | 5 (21) | 4 (13) | 0.430 |
| Rituximab + IVIG*, n (%)* | 3 (6) | 0 (0) | 3 (10) | 0.117 |
| Bortezomib*, n (%)* | 8 (15) | 1 (4) | 7 (23) | 0.055 |
| **Index biopsy results** |  |  |  |  |
| Microcirculation inflammation (g>0 ± ptc>0), *n (%)* | 52 (95) | 22 (92) | 30 (97) | 0.408 |
| g score, *median (IQR)* | 2 (1-2) | 2 (0-2) | 2 (1-2) | 0.458 |
| ptc score, *median (IQR)* | 2 (1-2) | 2 (1-2) | 2 (1-2) | 0.210 |
| g+ptc score, *median (IQR)* | 3 (2-4) | 3 (2-4) | 3 (2-4) | 0.562 |
| Transplant glomerulopathy (cg>0), *n (%)* | 40 (73) | 19 (79) | 21 (68) | 0.345 |
| cg score, *median (IQR)* | 2 (0-3) | 2 (1-3) | 1 (0-3) | 0.108 |
| Linear C4d+ in PTC, *n (%)* | 24 (44) | 10 (42) | 14 (45) | 0.931 |
| C4d score, *median (IQR)* | 0 (0-2) | 0 (0-2) | 0 (0-2) | 0.935 |
| Histologic criteria of acute / active ABMR*, n (%)* | 17 (31) | 6 (25) | 11 (36) | 0.404 |
| Histologic criteria of chronic / active ABMR*, n (%)* | 38 (69) | 18 (75) | 20 (65) | 0.404 |
| Concurrent TCMR*, n (%)* | 20 (36) | 9 (38) | 11 (35) | 0.877 |
| Borderline lesion*, n (%)* | 12 (22) | 5 (21) | 7 (23) | n.a. |
| IA or IB*, n (%)* | 4 (7) | 2 (8) | 2 (7) | n.a. |
| IIA*, n (%)* | 3 (6) | 1 (4) | 2 (7) | n.a. |
| Chronic TCMR*, n (%)* | 1 (2) | 1 (4) | 0 (0) | n.a. |
| Concurrent GN*, n (%)* | 9 (16) | 7 (29) | 2 (7) | 0.024 |
| IgA nephropathy, *n (%)* | 6 (11) | 4 (17) | 2 (6) | n.a. |
| Immune-complex GN (e.g. MPGN), *n (%)* | 3 (5) | 3 (13) | 0 (0) | n.a. |
| Thrombotic microangiopathy*, n (%)* | 3 (6) | 2 (8) | 1 (3) | 0.408 |

**^a^**Before 2009, pre-sensitized patients were defined as having a CDC-PRA ≥ 40%. ^b^Refers to percent of patients that underwent Luminex testing before transplantation, which was available since 2009 at our center and where the specificities of pre-Tx DSA were documented. In three patients with verified DSA before transplantation, specificities were not documented. **^c^**Four patients had a positive CDC-XM and underwent peri-transplant XM-conversion with immunoadsorption according to our center-protocol.

**Abbreviations:** ABMR, antibody-mediated rejection; ATG, anti-thymocyte globulin; iBx, index biopsy; CD3, cluster of differentiation 3; CDC-PRA, complement-dependent cytotoxicity-panel-reactive antibodies; cg, transplant glomerulopathy; CNI, calcineurin inhibitor; DSA, donor-specific antibody; g, glomerulitis; EOS, end of study; GN, glomerulonephritis; HLA, human leukocyte antigen; IA, immunoadsorption; IL-2, interleukin-2; IQR, interquartile range; IVIG, intravenous immunoglobulins; MFI, mean fluorescence intensity; MMF, mycophenolate mofetil; MPA, mycophenolic acid; MPGN, membranoproliferative glomerulonephritis; mTORi, inhibitor of mammalian target of rapamycin; n.a., not applicable; PLEX, plasma exchange; ptc, peritubular capillaritis; PTC, peritubular capillaries; TCMR, T cell-mediated rejection; Tx, transplantation, XM, crossmatch.

**Supplementary Table S3: *De novo* DSA specificities in 14 /17 subjects with known pre-transplant DSA specificities.**

| Subject ID^a^ | Pre-transplantation  specificities (MFI) | Post-transplantation (index biopsy)  specificities (MFI) |
| --- | --- | --- |
| 2 | Cw6 (13,300) | Cw6 (15,230) |
|  | B60 (5,700) |  |
|  | DR13 (4,300) |  |
| 9 | B8 Bw6 (500) | DQ2 (8,615) |
| 19 | DQ6 (900) | DQ6 (4,843) |
|  |  | DR51 (13,024) |
| 28 | A25 (15,000) | A25 (5,879) |
| 30 | B8 Bw6 (15,000) | B8 Bw6 (2,817) |
|  | DQ7 (800) | DQ7 (2,428) |
|  | Cw7 (700) | DQ9 (7,769) |
| 33 | DR14 (4,100) | DR14 (2,949) |
|  | DQ5 (7,000) | DQ5 (16,747) |
|  |  | DR52 (3,107) |
| 35 | DQ7 (2,000) | DQ7 (7,530) |
| 41 | Negative | DP13 (3,928) |
| 42 | B60 (4,000) | DQ6 (15,726) |
|  | B13 (2,500) |  |
|  | Cw6 (800) |  |
| 47 | DQ7 (900) | DQ7 (18,167) |
|  | A24 (1,500) |  |
|  | B44 (3,000) |  |
| 54 | Negative | DR51 (6,538) |
| 55 | A1 (12,000) | A1 (3,177) |
|  | DQ6 (1,500) |  |
| 60 | A33 (8,000) | A33 (5,472) |
|  | DQ7 (10,000) | DQ7 (16,059) |
| 64 | DQ6 (700) | DQ6 (2,755) |
|  | B7 (700) |  |
|  | DR1 (600) |  |

^a^Pre-transplant Luminex testing was available for 17/70 (24%) of patients. Three patients had an unknown pre-transplant DSA specificity. DSA highlighted in red are considered as *de novo* DSA.

**Supplementary Table S4**

| **Parameters** | **Estimate** | **95% confidence interval** | | **p-value** |
| --- | --- | --- | --- | --- |
| **Antibody-verified cohort (n=55)** |  | **lower** | **upper** |  |
| -12 to 12 months LME model | | | | |
| Intercept at iBx [ml/min/1.73m^2^] | 43.7 | 40.4 | 47.0 | <0.001 |
| eGFR slope pre-iBx [ml/min/1.73m^2^] | -8.6 | -11.9 | -5.3 | <0.001 |
| eGFR slope post-iBx [ml/min/1.73m^2^] | -6.7 | -9.8 | -3.6 | <0.001 |
| -12 to 24 months LME model | | | | |
| Intercept at iBx [ml/min/1.73m^2^] | 43.4 | 40.1 | 46.7 | <0.001 |
| eGFR slope pre-iBx [ml/min/1.73m^2^] | -9.0 | -12.4 | -5.6 | <0.001 |
| eGFR slope post-iBx [ml/min/1.73m^2^] | -5.6 | -7.7 | -3.6 | <0.001 |

**Abbreviations:** iBx, index biopsy; eGFR, estimated glomerular filtration rate; LME, linear mixed effects model

**Supplementary Table S5**

| **Parameters** | **Estimate** | **95% confidence interval** | | **p-value** |
| --- | --- | --- | --- | --- |
| **Total cohort (n=70)** |  | **lower** | **upper** |  |
| -12 to 12 months LME model | | | | |
| Intercept at iBx [ml/min/1.73m^2^] | 41.3 | 36.7 | 45.8 | <0.001 |
| Treatment | 2.8 | --3.0 | 8.6 | 0.344 |
| eGFR slope pre-iBx [ml/min/1.73m^2^] | -6.8 | -11.4 | -2.3 | 0.004 |
| Interaction Slope pre-iBx * Treatment | -2.8 | -8.6 | 3.0 | 0.336 |
| eGFR slope post-iBx [ml/min/1.73m^2^] | -5.5 | -9.9 | -1.2 | 0.013 |
| Interaction Slope post-iBx * Treatment | -2.5 | -8.0 | 3.1 | 0.386 |
| -12 to 24 months LME model | | | | |
| Intercept at iBx [ml/min/1.73m^2^] | 41.0 | 36.5 | 45.6 | <0.001 |
| Treatment | 2.4 | -3.4 | 8.2 | 0.411 |
| eGFR slope pre-iBx [ml/min/1.73m^2^] | -7.2 | -11.7 | -2.7 | 0.002 |
| Interaction Slope pre-iBx * Treatment | -3.3 | -9.1 | 2.5 | 0.262 |
| eGFR slope post-iBx [ml/min/1.73m^2^] | -4.8 | -7.7 | -1.9 | 0.002 |
| Interaction Slope post-iBx * Treatment | -1.2 | -5.0 | 2.6 | 0.535 |

**Abbreviations:** iBx, index biopsy; eGFR, estimated glomerular filtration rate; LME, linear mixed effects model
