# Supplementary figures and images for: Early Estimated Glomerular Filtration Rate Trajectories After Kidney Transplant Biopsy as a Surrogate Endpoint for Graft Survival in Late Antibody-Mediated Rejection
Source: Front Med (Lausanne). 2022 Apr 21;9:817127. doi: 10.3389/fmed.2022.817127 (PMC9069161; doi:10.3389/fmed.2022.817127)

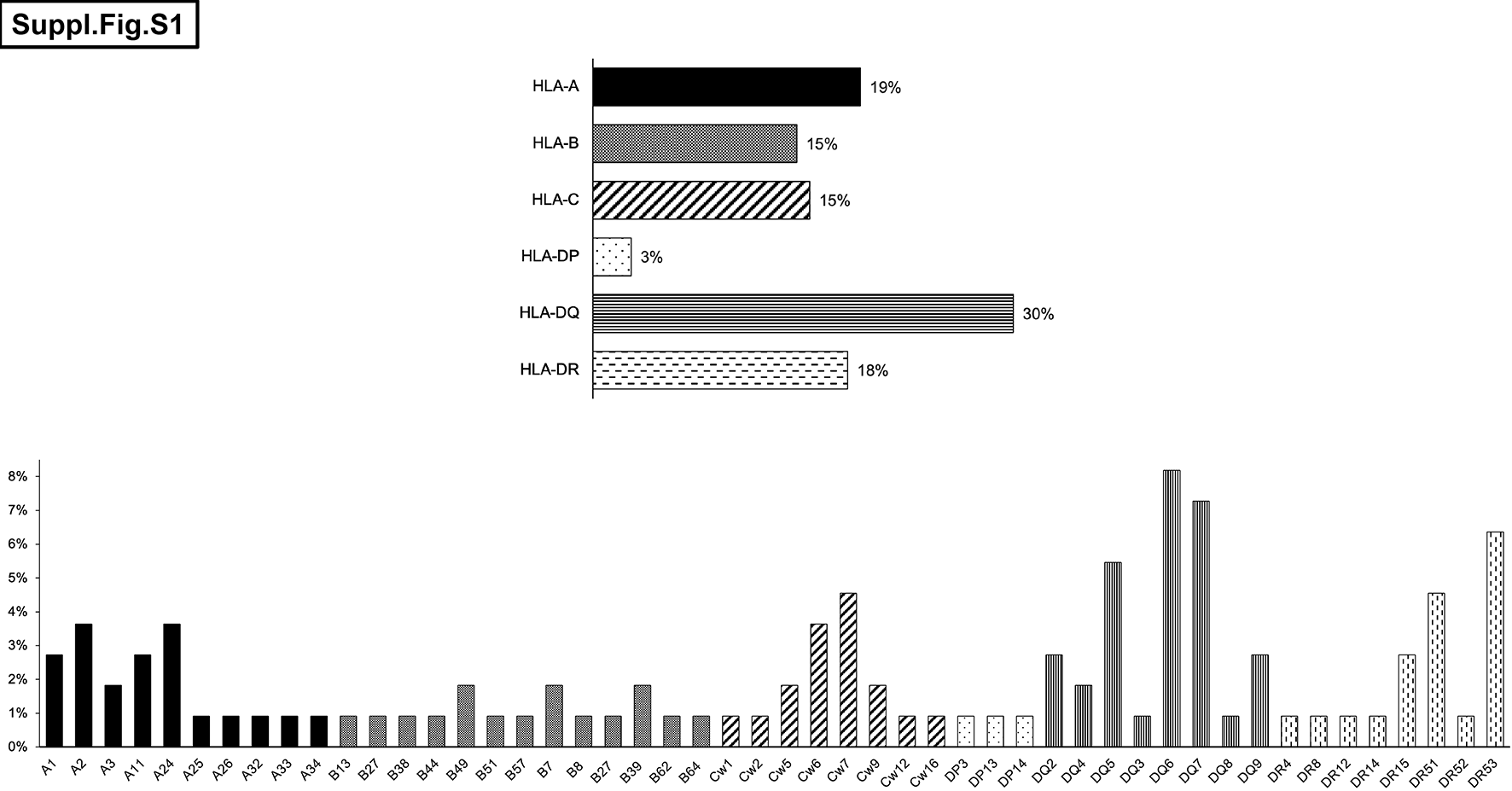

Supplement: Supplementary Figure 1 — Bar charts with percentages of the HLA class I and class II antigens that the corresponding DSA were directed against. Since some patients had > 1 DSA within either HLA class, percentages do not perfectly match with the given percentages of class I and/or class II DSA at the time of iBx in Table 2 and Supplementary Table 2. [file Image_1.TIF]

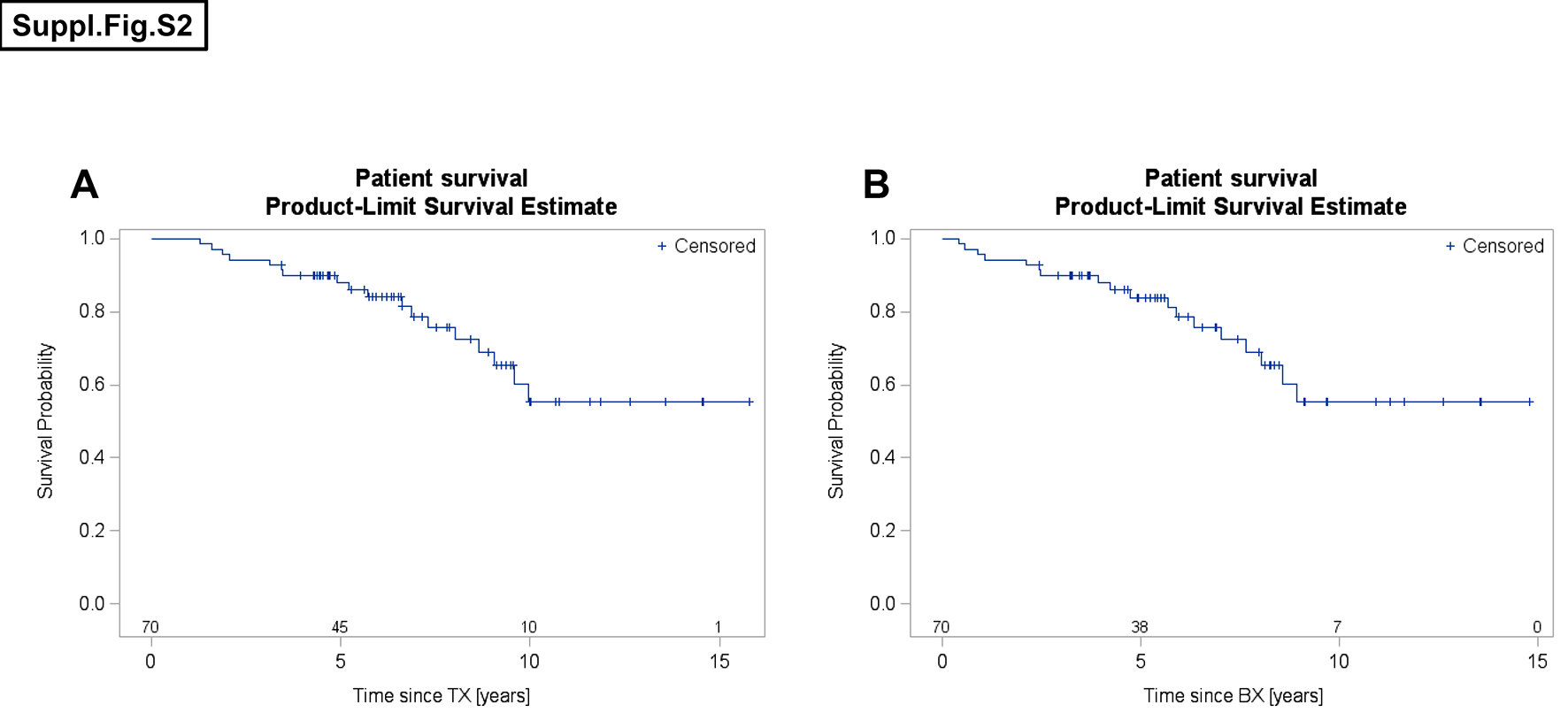

Supplement: Supplementary Figure 2 — Kaplan-Meier plots of the total cohort (N = 70) for patient survival since transplantation (A) and since index biopsy (B). [file Image_2.TIF]

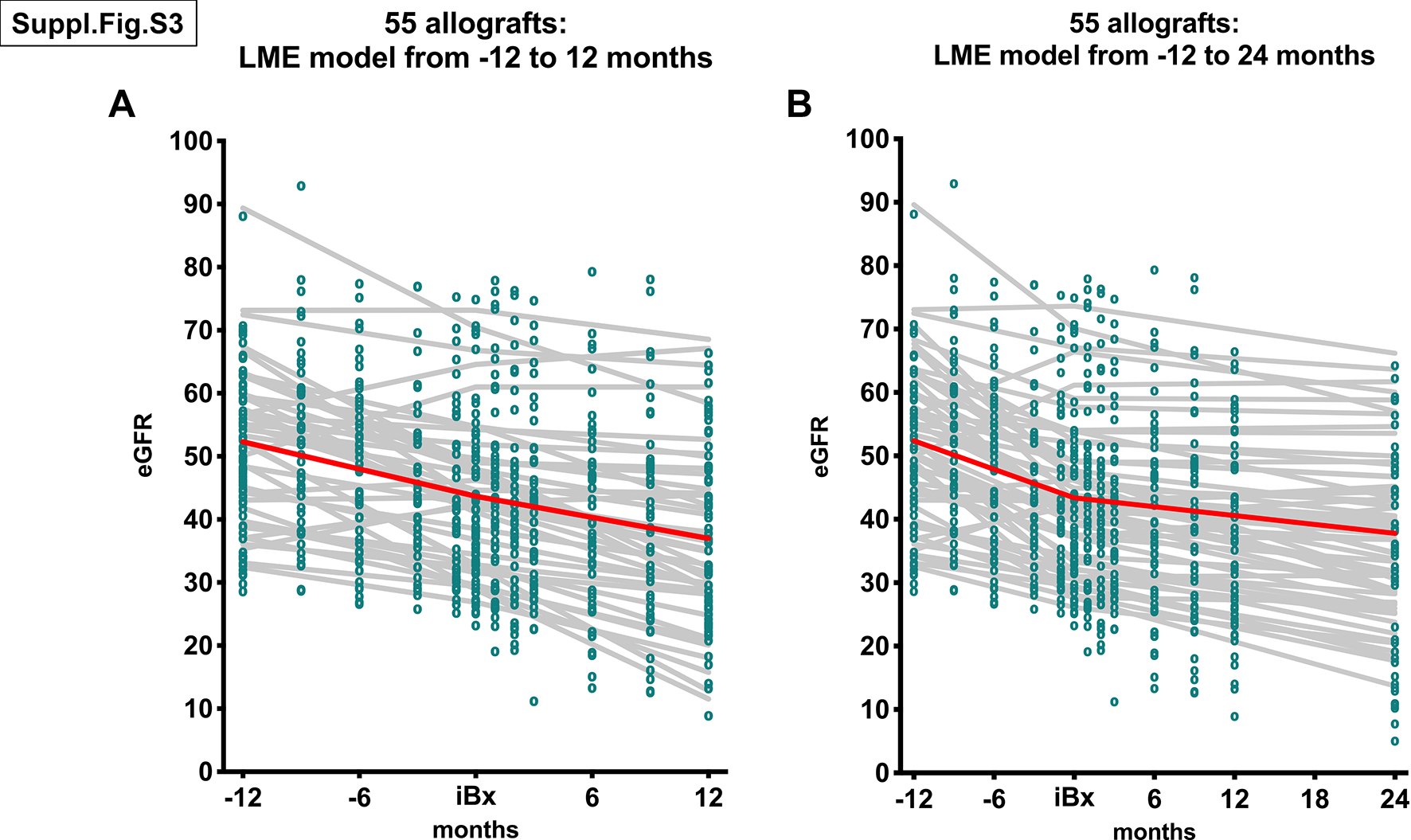

Supplement: Supplementary Figure 3 — It shows the single eGFR measurements (green dots), the individual eGFR trajectories (gray solid lines) as well as the overall trajectory (red solid line) of the antibody-verified cohort (N = 55). The linear mixed effects model (LME) was carried out over a course of 2 years (A) from −12 to 12 months with intercept at time of iBx and over the course of 3 years (B) from -12 to 24 months with intercept at the time of iBx. The shown overall trajectory is based on the fixed effects of the LME, whereas the individual trajectories also include the random effects. [file Image_3.TIF]

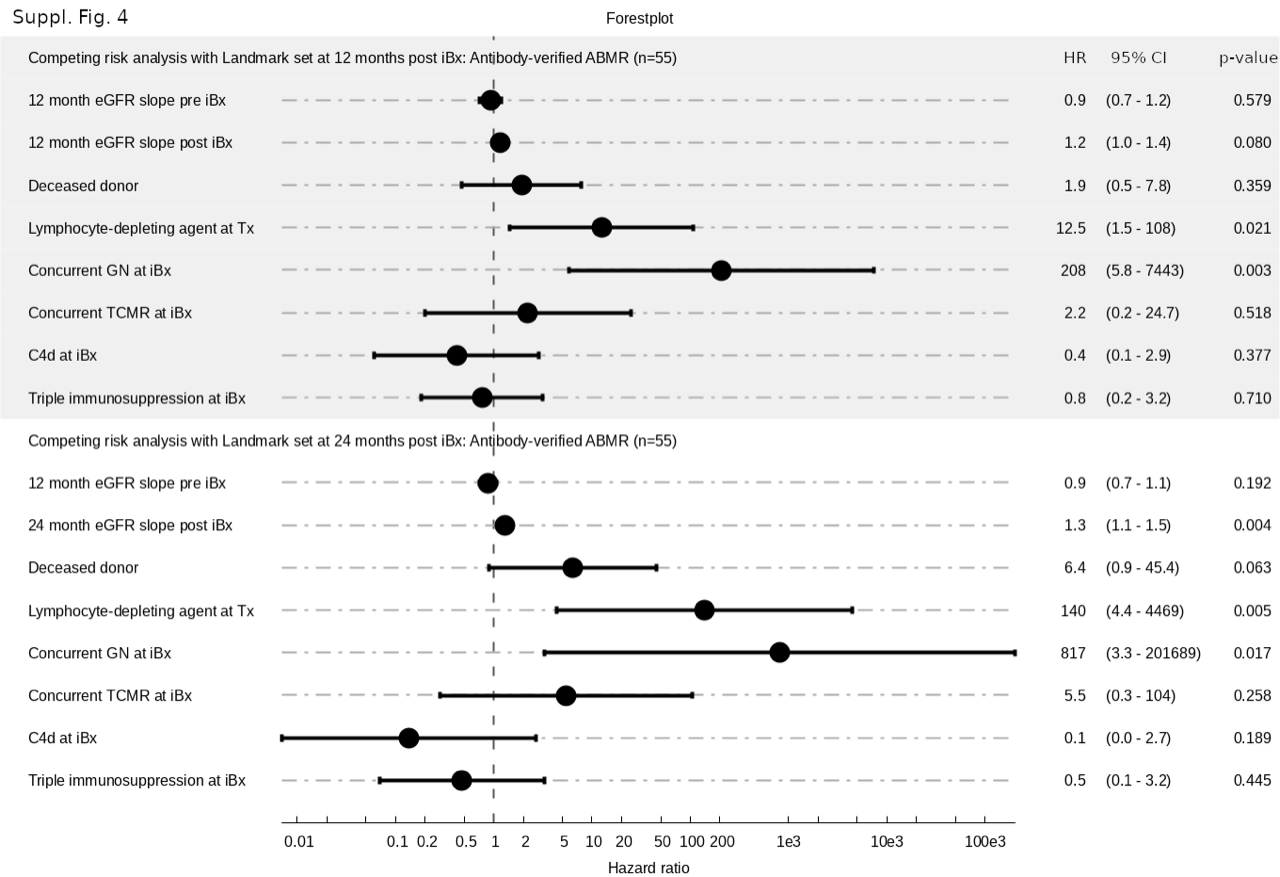

Supplement: Supplementary Figure 4 — Forest plots for the antibody-verified cohort (N = 55) with landmarks set at 12 or 24 months after iBx to avoid immortal time bias. Hazard ratios (HR’s) and their corresponding 95% confidence intervals (95% CI) are shown on the right. GN, glomerulonephritis; TCMR, T cell-mediated rejection. [file Image_4.TIF]
